# Checklist of national key protected wild plants on the Qinghai-Tibetan Plateau

**DOI:** 10.3897/BDJ.11.e103289

**Published:** 2023-05-16

**Authors:** Ronglian Chen, Faqi Zhang, Shilong Chen, Xiaofeng Chi

**Affiliations:** 1 University of Chinese Academy of Sciences, Beijing, China University of Chinese Academy of Sciences Beijing China; 2 Qinghai Provincial Key Laboratory of Crop Molecular Breeding, Northwest Institute of Plateau Biology, Chinese Academy of Sciences, Xining, China Qinghai Provincial Key Laboratory of Crop Molecular Breeding, Northwest Institute of Plateau Biology, Chinese Academy of Sciences Xining China

**Keywords:** Qinghai-Tibetan Plateau, national key protected wild plants, diversity characteristics, distribution pattern

## Abstract

**Background:**

Qinghai-Tibetan Plateau is a global biodiversity hotspot due to the unique geographical environment. However, there are few reports on the list of national key protected plants and the distribution pattern of their diversity in this area. Based on the flora and online database, this paper summarised the species diversity and distribution patterns of national key protected wild plants on the Qinghai-Tibet Plateau.

**New information:**

The results showed that there were 350 species of national key protected wild plants on the Qinghai-Tibetan Plateau, belonging to 72 families and 130 genera. Amongst them, 22 species were under class I protection, 328 species were under class II protection and 168 species were endemic to China. Its endangered status involves EW 1 species, CR 17 species, EN 90 species, VU 90 species, NT 30 species, LC 60 species and DD 62 species. Species diversity declined gradually from the southeast to the northwest with hotspots located within Sanjiang Valley subregion (ⅢE14a). The list of national key protected wild plants and their diversity and distribution patterns in the Qinghai-Tibetan Plateau can provide basic data for the conservation of regional biodiversity and the formulation of conservation strategies.

## Introduction

China is one of the most biodiverse countries in the world. There are approximately 483 families, 4,275 genera, 38,493 species and 7,507 subspecies of higher plants, of which approximately 18,000 species are endemic to China (Liu and Qin 2022). However, biodiversity in China is still under great pressure. On the one hand, species diversity is seriously affected by sharp population growth, rapid economic development and the degradation or even loss of natural ecosystems. On the other hand, excessive mining, alien species invasion and natural disasters severely damage the species diversity (Liu et al. 2020). The Chinese government attaches great importance to biodiversity conservation. Relevant laws and policies have been promulgated and the legal framework has been continuously improved. The State Council issued "the List of National Key Protected Wild Plants (First Batch)" in 1999, which includes 246 plant species (varieties) and eight plant categories (State Forestry Administration and the Ministry of Agriculture, P.R. China 1999). The protection of wild plants in China has undergone significant changes since the release of the list in 1999. Some endangered wild plants have been effectively protected and the degree of endangerment has reduced. However, significant changes have occurred in the protection of wild plant diversity in China over the past 20 years. Therefore, in September 2021, the National Forestry and Grassland Administration and the Ministry of Agriculture and Rural Affairs released "the List of National Key Protected Wild Plants (2021)" (hereinafter as *List* (2021)), which includes approximately 1,101 species (455 species and 40 categories) of wild plants, of which 54 species and four categories of wild plants are under first-class state protection and 401 species and 36 categories of wild plants under second-class state protection (State Forestry and Grassland Administration and the Ministry of Agriculture and Rural Affairs, P. R. China 2021). The 15^th^ UN Biodiversity Conference (COP15), held in October 2021, discussed the Post-2020 Global Biodiversity Framework, with the main objective of responding to the continuing deterioration of biodiversity worldwide. Therefore, strengthening the protection of wild plants to reverse the decline of wild populations and extinction of species was one of the key topics. In this context, based on the *List* (2021), it is necessary to investigate and sort the species, family and genus composition, endemism, endangered status, geographical distribution and other characteristics of national key protected wild plants in specific areas, especially in ecologically fragile areas, to provide important references for in situ protection, ex situ protection and standardised use of species.

Known as the "roof of the world" and the "third pole", the Qinghai-Tibetan Plateau is the highest and youngest plateau in the world. With diverse vegetation types and complex floristic components, the Qinghai-Tibetan Plateau is the most biodiverse region on Earth. It is also a vulnerable area of ecosystem and is sensitive to climate change. This region contains three global biodiversity hotspots: the Himalayas, mountains of Southwest China and Indo-Burma (Myers et al. 2000). The unique natural environment and strong changes in the historical geological environment make the Qinghai-Tibetan Plateau possess many complex fragmented habitats and rich species. According to the second Qinghai-Tibet Scientific Survey data, there are 14,634 vascular plant species on the Qinghai-Tibetan Plateau, accounting for 45.8% of vascular plants in China (Fu et al. 2021). Amongst these, 3,764 species of seed plants endemic to the Qinghai-Tibetan Plateau were included (Yu et al. 2018).

With the intensification of global climate change and owing to the vulnerability and sensitivity of the Qinghai-Tibetan Plateau to climate change and increasing human activities, the biodiversity of this region is facing a severe threat (Liu et al. 2022) and various wild plants are highly endangered. However, there are only a few reports on the diversity and distribution patterns of national key protected plants on the Qinghai-Tibetan Plateau. Therefore, based on the *List* (2021), this study preliminarily sorted out the species of national key protected wild plants in the Qinghai-Tibetan Plateau Region, analysed their diversity, endangered status and endemic status and explored their distribution patterns, hoping to provide a reference and basis for the study of national key protected wild plants on the Qinghai-Tibetan Plateau.

## Materials and methods

### Determination of plant list

With reference to the Qinghai-Tibetan Plateau boundary data (Zhang 2019), plants in the *List* (2021), distributed only in the Qinghai-Tibetan Plateau Region, were collected. First, based on the Flora of China (Wu et al. 1994), specimen information was obtained from the National Specimen Information Infrastructure (NSII, http://www.nsii.org.cn/2017) and the Chinese Virtual Herbarium (CVH, https://www.cvh.ac.cn/index.php). Combined with field scientific survey records collected in recent years, the family and species lists, as well as county-level distribution of key protected wild plants on the Qinghai-Tibet Plateau were, sorted out. Threatened status and endemism of species were confirmed from the IUCN Red List of Threatened Species 2021 (IUCN Red List of Threatened Species), China Red List of Biodiversity (Higher Plants Volume) (Ministry of Environmental Protection of the People’s Republic of China and Chinese Academy of Sciences 2018). Based on cataloguing, diversity characteristics of national key protected wild plants on the Qinghai-Tibetan Plateau were analysed and summarised, including family and genus composition characteristics, protection level, endemism, endangered status and geographical distribution characteristics. Floristic division scales of Qinghai-Tibetan Plateau were according to the Floristics of seed plants from China (Wu et al. 2010). The summarised checklist was arranged in order of Bryophyta, Lycopodiophyta, Pteridophyta, Gymnospermae and Angiospermae and the Angiospermae genera are arranged according to the APG IV system (The Angiosperm Phylogeny Group et al. 2016).

### Statistical analysis

According to *List* (2021), the basic characteristics of key protected plants distributed on the Qinghai-Tibetan Plateau were summarised, including the quantity and proportion of families, genera and species, protection level classification, quantity of endangered plants and endemic species to China.

### Diversity pattern analysis

County-scale distribution data of key protected plants on the Tibetan Plateau were collected. The Qinghai-Tibet Plateau covers 221 county-level administrative regions (including municipal districts), including 45 in Qinghai Province, 74 in Tibet Autonomous Region, 48 in Sichuan Province, 28 in Gansu Province, 12 in Yunnan Province and 14 in Xinjiang Uygur Autonomous Region. The data collection sources of species distribution at the county level mainly included: (1) Annals and monographs, such as Flora of China (Wu et al. 1994), Flora of Qinghai (Northwest Institute of Plateau Biology, Chinese Academy of Sciences 1996), Flora of Sichuan (Volumes 1–21) (Editorial Committee of Flora Sichuanica 1981), Flora of Yunnan (Kunming Institute of Botany, Chinese Academy of Sciences 1977), Flora of Tibet (Wu 1983), Flora of Kunlun (Wu 2014), Vascular Plants and their Ecogeographical Distribution in the Qinghai-Tibet Plateau (Wu 2008) and the Second Survey of National Key Protected Wild Plant Resources in Gansu Province (Zhu and Kou 2020); (2) Online databases, such as National Specimen Information Infrastructure (NSII, http://www.nsii.org.cn/2017) and the Chinese Virtual Herbarium (CVH, https://www.cvh.ac.cn/index.php); (3) Field survey data of the Qinghai-Tibetan Plateau.

According to species in each county and floristic division, ArcGIS 10.6 was used to plot the diversity pattern of national key protected wild plants on the Qinghai-Tibet Plateau at county and floristic division scales.

## Checklists

### Checklist of National Key Protected Wild Plants on the Qinghai-Tibetan Plateau

#### Leucobryum juniperoideum

C. Müller, 1845

4BB82F76-8B56-59CC-8536-ED7D9D0A52DE

##### Conservation status

LC

##### Distribution

East Asia, Europe

#### Sphagnum multifibrosum

X.J. Li & M. Zang, 1984

72C067D0-749A-5F04-B034-24048B0BDC3A

##### Conservation status

LC

##### Distribution

China

#### Sphagnum squarrosum

Crome, 1803

FB7CE3D7-6B5D-5D55-BC7A-E9A88DDDFB26

##### Conservation status

LC

##### Distribution

East Asia, Europe, Oceania, North America

#### Takakia ceratophylla

(Mitt.) Grolle, 1963

D5A08AA8-1E31-541D-8D4E-DD1F1378BE1C

##### Conservation status

VU

##### Distribution

China, North America

#### Takakia lepidozioides

S. Hatt. & Inoue, 1958

97351AEF-8C47-5872-9B5D-268FA8BC4682

##### Conservation status

EN

##### Distribution

China, Japan, North America

#### Huperzia appressa

(Desv.) Á. Löve & D. Löve, 1961

BD5F3255-A356-5127-9D4B-0784B9CC3B56

##### Conservation status

DD

##### Distribution

Asia, Europe, North America

#### Huperzia bucahwangensis

Ching, 1981

13BF6753-B236-5FAA-A84E-6C2F2F15F508

##### Conservation status

DD

##### Distribution

China

#### Huperzia chinensis

Ching, 1981

E66BF753-9244-59D7-947C-DD19C4A74DCD

##### Conservation status

NT

##### Distribution

China

#### Huperzia crispata

Ching, 1981

E3672F1A-557A-5491-83E8-89F0BDEE9EBB

##### Conservation status

VU

##### Distribution

China

#### Huperzia delavayi

Ching, 1981

3F9CAD32-87D9-50CB-AF3B-093721F46BF8

##### Conservation status

DD

##### Distribution

China

#### Huperzia dixitiana

P. Mondal & R.K. Ghosh, 1995

446F2C3E-C089-52FA-AE13-D1530994373B

##### Conservation status

DD

##### Distribution

China

##### Notes

Endemic to Qinghai-Tibetan Plateau

#### Huperzia emeiensis

Ching & H.S. Kung, 1981

C07C8A07-3055-5DC4-A6F9-50CF0EE55896

##### Conservation status

DD

##### Distribution

China

#### Huperzia herteriana

(Kumm.) T. Sen & U. Sen, 1978

30509449-4625-594B-9D8F-5B16A5C5F97A

##### Conservation status

DD

##### Distribution

China, Bhutan, India, Nepal

#### Huperzia kangdingensis

Ching, 1981

FA76D619-275C-5447-9857-B6015B5570C6

##### Conservation status

DD

##### Distribution

China

#### Huperzia lajouensis

Ching, 1981

1F516D1B-2787-5E88-8389-BE994B6A19B5

##### Conservation status

DD

##### Distribution

China

##### Notes

Endemic to Qinghai-Tibetan Plateau

#### Huperzia liangshanica

(H.S. Kung) Ching & H.S. Kung, 1981

AE8BD48A-2CC0-5D94-B641-5DF6C80F7CD1

##### Conservation status

DD

##### Distribution

China

#### Huperzia quasipolytrichoides

(Hayata) Ching, 1981

FB04E19B-0E65-5052-937F-1FC38AB0F29B

##### Conservation status

VU

##### Distribution

China, Japan

#### Huperzia rubicaulis

S.K. Wu & X. Cheng, 1985

01F1DCA9-29A1-5B4E-895B-8BEC76C37C75

##### Conservation status

DD

##### Distribution

China

##### Notes

Endemic to Qinghai-Tibetan Plateau

#### Huperzia selago

(L.) Bernh. ex Schrank & Martius, 1829

2191DFF7-0C0C-5FBD-ADAD-7FFF009708D1

##### Conservation status

LC

##### Distribution

America, Asia, Europe, Pacific islands

#### Huperzia serrata

(Thunb.) Trevis, 1875

83460015-BB3A-5FF9-9DAD-D45F79BA35E1

##### Conservation status

EN

##### Distribution

East Asia, the Pacific, Russia, Oceania, Central America

#### Huperzia sutchueniana

(Herter) Ching, 1981

7B40967C-F55C-5FE1-9EA0-C73B1B13EB17

##### Conservation status

DD

##### Distribution

China

#### Huperzia tibetica

Ching, 1981

41F1DB4E-1120-57DF-BC70-5A41083A4B3B

##### Conservation status

DD

##### Distribution

China

##### Notes

Endemic to Qinghai-Tibetan Plateau

#### Phlegmariurus cancellatus

(Spring) Ching, 1982

E14FA1F4-4F57-515B-8202-8C3122D781EE

##### Conservation status

DD

##### Distribution

China, India, Bhutan

#### Phlegmariurus fargesii

(Herter) Ching, 1982

2C8505A6-3199-55B3-B3D1-26BE6EEBE9DA

##### Conservation status

DD

##### Distribution

China, Japan

#### Phlegmariurus hamiltonii

(Spreng.) A. Löve & D. Löve, 1977

EC6CAE96-290D-5DCB-86D3-191741419AFD

##### Conservation status

DD

##### Distribution

China, India, Nepal, Bhutan, Myanmar

#### Phlegmariurus nylamensis

(Ching & S.K. Wu) H.S. Kung & Li Bing Zhang, 1999

85A44513-D212-5B41-8846-07B7131FC395

##### Conservation status

DD

##### Distribution

China

##### Notes

Endemic to Qinghai-Tibetan Plateau

#### Phlegmariurus pulcherrimus

(Hook. & Grev.) Á. Löve & D. Löve, 1977

AE5707BB-BD2B-58C6-A840-8C4E20BBAFCB

##### Conservation status

DD

##### Distribution

China, Bhutan, India

#### Phlegmariurus squarrosus

Á. Löve & D. Löve, 1977

E4D96807-86CF-5137-A77F-9BB7E2E103D2

##### Conservation status

NT

##### Distribution

China, India, Nepal, Myanmar, Thailand, Vietnam, Laos, Cambodia, Bangladesh, Sri Lanka, Malaysia, Philippines, Polynesia, Madagascar

#### Phlegmariurus subulifolius

(Wall. ex Hook. & Grev.) S.R. Ghosh, 2009

A6A5CD9F-A6BA-50BC-A95C-5097F869B3AD

##### Conservation status

DD

##### Distribution

China, India, Nepal

#### Phlegmariurus yunnanensis

Ching, 1982

38A8AAAE-3ACD-5CC2-A9D2-3E058A8E2CE0

##### Conservation status

DD

##### Distribution

China

##### Notes

Endemic to Qinghai-Tibetan Plateau

#### Isoetes hypsophila

Handel-Mazz., 1923

C6668A88-863F-50C4-B551-D7787E178D9D

##### Conservation status

VU

##### Distribution

China

##### Notes

Endemic to Qinghai-Tibetan Plateau

#### Isoetes shangrilaensis

Xiang Li, Yuqian Huang, X. Dai & Xing Liu, 2019

1C08D791-698D-5926-8BCF-BD29124821A2

##### Conservation status

DD

##### Distribution

China

##### Notes

Endemic to Qinghai-Tibetan Plateau

#### Angiopteris esculenta

Ching, 1940

9F0BA0CA-977D-55AF-BD30-258110E6A769

##### Conservation status

DD

##### Distribution

China

#### Angiopteris wallichiana

C. Presl, 1845

3F6DE518-1706-59D6-9182-2D93A10706E8

##### Conservation status

DD

##### Distribution

China, India, Nepal

#### Angiopteris fokiensis

Hieron., 1919

4A73F901-FFA6-5921-9425-F849FDA3599F

##### Conservation status

DD

##### Distribution

China, Japan

#### Cibotium barometz

(L.) J. Smith,1842

9F8A55BB-11B8-52EC-8F82-E3BF3767C27C

##### Conservation status

LC

##### Distribution

China, India, Indonesia, Japan, Malaysia, Myanmar, Thailand, Vietnam

#### Sphaeropteris brunoniana

(Hook.) R.M. Tryon, 1970

6A771A37-1728-5B1C-86C0-6ABB2EF43B2D

##### Conservation status

EN

##### Distribution

China, Bhutan, Nepal, India, Bangladesh, Myanmar, Vietnam

#### Gymnosphaera andersonii

(J. Scott ex Bedd.) Ching & S.K. Wu, 1983

87F40718-DE94-5797-9C07-2450B097112C

##### Conservation status

DD

##### Distribution

China

#### Gymnosphaera khasyana

(Moore ex Kuhn) Ching, 1984

19C5084A-41D7-57F8-9763-0821E13AC04E

##### Conservation status

DD

##### Distribution

China, India, Myanmar

#### Gymnosphaera metteniana

(Hance) Tagawa, 1951

976617BE-0E21-5CC6-8269-BB150B68133A

##### Conservation status

DD

##### Distribution

China, Japan

#### Alsophila costularis

Baker, 1906

C978AD30-5FA3-5400-A009-3DDB96F35D0B

##### Conservation status

LC

##### Distribution

China, Bhutan, India, Vietnam, Myanmar, Bangladesh

#### Alsophila spinulosa

(Hook.) R.M. Tryon, 1970

5F1E300D-25EE-5F17-AD4C-D436C7F67DA4

##### Conservation status

NT

##### Distribution

China, Japan, Vietnam, Cambodia, Thailand, Myanmar, Bangladesh, Bhutan, Nepal, India

#### Cystopteris chinensis

(Ching) X.C. Zhang & R. Wei, 2014

7D5D53FE-6DDF-5D43-A7DF-44A6F056E50C

##### Conservation status

EW

##### Distribution

China

#### Podocarpus macrophyllus

(Thunb.) Sweet, 1818

4067E586-4679-5CF1-ADCF-D5C3A586AE76

##### Conservation status

LC

##### Distribution

China, Japan

#### Podocarpus neriifolius

D. Don, 1824

3872D1F6-EA45-5989-B7AA-303D0AEA3956

##### Conservation status

LC

##### Distribution

China, Nepal, India, Bhutan, Myanmar, Vietnam, Laos, Indonesia, Malaysia

#### Cupressus chengiana

S.Y. Hu, 1964

9DB5FAEB-F13A-55CF-8B81-07ABFA8A082A

##### Conservation status

VU

##### Distribution

China

##### Notes

Endemic to Qinghai-Tibetan Plateau

#### Cupressus gigantea

W.C. Cheng & L.K. Fu, 1975

313B375E-4E13-564C-A09E-F75FF91419F9

##### Conservation status

VU

##### Distribution

China

##### Notes

Endemic to Qinghai-Tibetan Plateau

#### Cupressus torulosa

D. Don, 1824

FE1E641C-D980-5CD8-AA7C-9199604813F9

##### Conservation status

LC

##### Distribution

China, India, Nepal, Bhutan

#### Taiwania cryptomerioides

Hayata, 1906

C56A749F-EF9E-5CA9-8502-C96BC96DB769

##### Conservation status

VU

##### Distribution

China

#### Thuja sutchuenensis

Franch., 1899

A5FD03FE-6800-5F62-9C58-23FFE94852FC

##### Conservation status

EN

##### Distribution

China

#### Cephalotaxus hainanensis

H.L. Li, 1954

F1BEE4F9-FEB9-526B-8A06-D6A3713A3B6C

##### Conservation status

EN

##### Distribution

China, Vietnam

#### Cephalotaxus griffithii

Hook. f., 1888

CEAF16AE-53E1-5C3B-BC58-DA07CF245AE5

##### Conservation status

EN

##### Distribution

China, Myanmar

#### Amentotaxus argotaenia

(Hance) Pilg., 1916

E9173B14-4903-5548-9FAF-6F47620161F1

##### Conservation status

NT

##### Distribution

China, Vietnam

#### Taxus contorta contorta

Griff., 1854

A53FDAEF-BAA5-54E4-B2F8-3E1827F4A9B8

##### Conservation status

EN

##### Distribution

China, India, Kashmir, Nepal, Pakistan

#### Taxus florinii

Spjut, 2007

B25D216D-F0F2-503B-B6FB-3A9C2513741F

##### Conservation status

DD

##### Distribution

China

#### Taxus wallichiana

Zucc., 1843

E11D5D3C-DD4D-5CA8-BB47-6005A59408E8

##### Conservation status

EN

##### Distribution

China, Bhutan, N India, Laos, Myanmar, Vietnam

#### Taxus wallichiana var. mairei

(Lemée & H. Lév.) L.K. Fu & Nan Li, 1997

B8C567EA-3E7B-5F16-BB17-4EBC038D35F5

##### Conservation status

VU

##### Distribution

China, India, Laos, Myanmar, Vietnam

#### Taxus wallichiana var. chinensis

(Pilg.) Florin,1948

59F18ED0-5DBE-5A27-9F63-02F2F7427009

##### Conservation status

VU

##### Distribution

China, Vietnam

#### Taxus yunnanensis

W.C. Cheng & L.K. Fu, 1975

6E1334C8-06E0-5E22-923D-5F32642AFDB6

##### Conservation status

DD

##### Distribution

China

#### Torreya fargesii

Franch., 1899

2B0FC13C-A416-517F-BEBC-DDCFCF78327F

##### Conservation status

VU

##### Distribution

China

#### Torreya yunnanensis

W.C. Cheng & L.K. Fu, 1975

9CD96765-1881-55BD-AB7D-D3E2A418D150

##### Conservation status

DD

##### Distribution

China

#### Abies chensiensis

Tiegh., 1892

71AC7C98-CA19-54AD-8970-AD6E8904D275

##### Conservation status

LC

##### Distribution

China

#### Keteleeria fortunei var. cyclolepis

(Flous) Silba, 1990

7A5BFFA7-28AF-5E12-8663-88AC5BB3A0E6

##### Conservation status

DD

##### Distribution

China

#### Keteleeria hainanensis

Chun & Tsiang, 1963

6D7E5BF9-BA92-5FC8-8810-6705F872F5B2

##### Conservation status

EN

##### Distribution

China

#### Picea neoveitchii

Mast., 1903

3E172DBE-AEDF-5353-9E91-6B6458D166D9

##### Conservation status

DD

##### Distribution

China

#### Pseudotsuga brevifolia

W.C. Cheng & L.K. Fu, 1975

3A1BB1B0-9E66-58F0-8CCA-58FEA5CE54C0

##### Conservation status

VU

##### Distribution

China

#### Pseudotsuga forrestii

Craib, 1920

635AF29B-9449-552C-8BA5-922F1AEDE1C5

##### Conservation status

VU

##### Distribution

China

##### Notes

Endemic to Qinghai-Tibetan Plateau

#### Pseudotsuga sinensis

Dode, 1912

D5909B2F-E4C5-5790-974A-3C4793965434

##### Conservation status

VU

##### Distribution

China

#### Saruma henryi

Oliv., 1889

9CD45E56-43EB-5356-AC7D-9AB4DF0131D3

##### Conservation status

EN

##### Distribution

China

#### Horsfieldia hainanensis

Merr., 1932

29B9AE1C-7A7D-5A19-92BB-64C180599440

##### Conservation status

DD

##### Distribution

China

#### Horsfieldia kingii

(Hook.f.) Warb., 1897

AA9FDAE3-55FB-58A4-BA50-CE0488192190

##### Conservation status

VU

##### Distribution

China, India, Bangladesh

#### Horsfieldia tetratepala

C.Y. Wu & W.T. Wang, 1957

615D7106-8378-519F-AFBC-68803FF9F936

##### Conservation status

DD

##### Distribution

China

#### Houpoea officinalis

(Rehder & E.H. Wilson) N.H. Xia & C.Y. Wu, 2008

29A2D368-23EE-552D-B81F-9AE53D22966F

##### Conservation status

NT

##### Distribution

China

#### Houpoea rostrata

(W.W. Sm.) N.H. Xia & C.Y. Wu, 2008

C050C40F-1025-5F9F-9375-CBFB42301FB3

##### Conservation status

VU

##### Distribution

China, Myanmar

#### Lirianthe henryi

(Dunn) N.H. Xia & C.Y. Wu, 2008

A014B101-13B0-5528-9747-E8C449FDF301

##### Conservation status

EN

##### Distribution

China, Myanmar, Thailand

#### Alcimandra cathcartii

Dandy, 1927

4D4F7CF1-7B7C-5067-A7C4-A040A72855B6

##### Conservation status

LC

##### Distribution

China, India

#### Liriodendron chinense

(Hemsl.) Sarg., 1903

138CC19A-AB5B-5794-8FFC-0819C59AD5EA

##### Conservation status

NT

##### Distribution

China, Vietnam

#### Michelia wilsonii

Finet & Gagnep., 1906

3158AD11-9177-5B44-9ABD-BE5852507582

##### Conservation status

EN

##### Distribution

China

#### Oyama sinensis

(Rehder & E.H. Wilson) N.H. Xia & C.Y. Wu, 2008

0F012287-D121-5C84-95AA-BE2519BE661A

##### Conservation status

VU

##### Distribution

China

#### Oyama wilsonii

(Finet & Gagnep.) N.H. Xia & C.Y. Wu, 2008

2A20AE74-8F9D-5243-9DC8-0F4B7CFD335C

##### Conservation status

NT

##### Distribution

China

#### Cinnamomum longepaniculatum

(Gamble) N. Chao ex H.W. Li, 1975

960CCA61-07AB-53FE-ADB1-F9E0D1B424A0

##### Conservation status

NT

##### Distribution

China

#### Machilus nanmu

(Oliv.) Hemsl., 1891

6FC32F7C-E78E-5A8B-A930-DA8CFEC1653F

##### Conservation status

EN

##### Distribution

China

#### Phoebe bournei

(Hemsl.) Yang, 1945

9699AE9C-754C-54E2-9251-ADE2925B89EE

##### Conservation status

NT

##### Distribution

China

#### Phoebe hui

Cheng ex Yang, 1945

67E6B16F-2939-57DA-BB9C-06361D998A88

##### Conservation status

NT

##### Distribution

China

#### Phoebe zhennan

S.K. Lee & F.N. Wei, 1979

CEAEF8F2-69AE-5B63-B23B-936A912B479B

##### Conservation status

VU

##### Distribution

China

#### Scheuchzeria palustris

Linnaeus, 1753

43473F8E-2B92-5403-9174-A9D9596D50C5

##### Conservation status

VU

##### Distribution

China, Japan, Korea, Mongolia, Russia, SW Asia, Europe, North America

#### Ottelia acuminata

(Gagnep.) Dandy, 1934

5FE3ED64-C998-5A0D-9E8F-CC3F8B40264F

##### Conservation status

VU

##### Distribution

China

#### Ottelia acuminata var. crispa

(Hand.-Mazz.) H. Li, 1981

06EAFF10-0D1F-5864-961B-8F0CB763403C

##### Conservation status

NT

##### Distribution

China

##### Notes

Endemic to Qinghai-Tibetan Plateau

#### Acanthochlamys bracteata

P.C. Kao, 1980

F18372F2-1CBF-5E4B-AA1A-BF1EA6334B90

##### Conservation status

VU

##### Distribution

China

##### Notes

Endemic to Qinghai-Tibetan Plateau

#### Paris axialis

H. Li, 1984

981C4069-7463-59FE-9836-515B4BBD7FBE

##### Conservation status

VU

##### Distribution

China

#### Paris bashanensis

F.T. Wang & Tang, 1978

F06826D0-2C46-5E9D-8FD5-E5458F8CD8DE

##### Conservation status

NT

##### Distribution

China

#### Paris delavayi

Franch., 1898

68BAF551-E50D-5EFD-B730-723E1047DB98

##### Conservation status

VU

##### Distribution

China, Vietnam

#### Paris dulongensis

H. Li & Kurita, 1992

4B41B3FD-4358-546A-81E5-71EEC72B50B6

##### Conservation status

CR

##### Distribution

China

##### Notes

Endemic to Qinghai-Tibetan Plateau

#### Paris fargesii

Franch., 1898

D522E8A6-9570-5DF8-9C47-89B48FB981EA

##### Conservation status

NT

##### Distribution

China, Vietnam

#### Paris fargesii var. petiolata

(Baker ex C.H. Wright) F.T. Wang & Tang, 1978

7657C62F-413A-58E1-8BC3-4D5832D39CF2

##### Conservation status

EN

##### Distribution

China

#### Paris forrestii

(Takht.) H. Li, 1984

DEA98A56-73A1-587B-9A1F-6D2AD0CBD8E5

##### Conservation status

EN

##### Distribution

China, Myanmar

#### Paris mairei

H. Lév., 1912

347A45E0-5305-5719-AEAF-588129C4608A

##### Conservation status

EN

##### Distribution

China

#### Paris marmorata

Stearn, 1956

C3DA1B95-5005-5A9C-8D67-F11155412380

##### Conservation status

DD

##### Distribution

China, Bhutan, India, Nepal

#### Paris polyphylla

Sm., 1813

30B3F09D-8660-565C-998C-55A46C316602

##### Conservation status

VU

##### Distribution

China, Bhutan, India, Laos, Myanmar, Nepal, Thailand, Vietnam

#### Paris polyphylla var. alba

H. Li & R.J. Mitch., 1986

4266AB59-9C29-5DD0-B610-AC837CB19ED8

##### Conservation status

VU

##### Distribution

China

#### Paris polyphylla var. chinensis

(Franch.) H. Hara, 1969

42509061-AF99-56FE-AFE1-741E2998AACF

##### Conservation status

VU

##### Distribution

China, Laos, Myanmar, Thailand, Vietnam

#### Paris polyphylla var. latifolia

F.T. Wang & C.Yu Chang, 1978

5726D38D-98DC-5BB6-9B44-36325798C64F

##### Conservation status

LC

##### Distribution

China

#### Paris polyphylla var. stenophylla

Franch., 1888

5D5ABC4D-6034-5493-BDCC-BEFAC650D7FB

##### Conservation status

NT

##### Distribution

China, Bhutan, India, Myanmar, Nepal

#### Paris polyphylla var. thibetica

(Franch.) H. Hara, 1969

224D1CE5-9953-58D9-B1F3-632FBD6A24A0

##### Conservation status

NT

##### Distribution

China

#### Paris polyphylla var. yunnanensis

(Franch.) Hand.-Mazz., 1936

4E64B2A8-54BF-505C-BADD-9B7E1B8B840B

##### Conservation status

NT

##### Distribution

China, India, Myanmar

#### Paris rugosa

H. Li & S. Kurita, 1992

E406772E-0F83-5D1D-ADAD-FFA099134597

##### Conservation status

EN

##### Distribution

China

#### Paris thibetica

Franch., 1888

BAEB5982-4379-539A-8865-5605197999E3

##### Conservation status

NT

##### Distribution

China, Bhutan, Myanmar, India

#### Paris thibetica var. apetala

Hand.-Mazz., 1925

9FD0C1D2-DCE5-59CC-9F95-4342FAD07D3D

##### Conservation status

DD

##### Distribution

China, Bhutan, Myanmar, India

#### Paris wenxianensis

Z.X. Peng & R.N. Zhao, 1986

41F15B7B-3208-5B12-815D-359CB4EF46FB

##### Conservation status

CR

##### Distribution

China

#### Cardiocrinum cathayanum

(E.H. Wilson) Stearn, 1948

45FAAA9A-F6A0-5DDF-8043-7ED1B09C650D

##### Conservation status

LC

##### Distribution

China

#### Fritillaria cirrhosa

D. Don, 1825

172C79FE-7173-5D60-BE76-E93FA5FBDF24

##### Conservation status

NT

##### Distribution

China, Bhutan, India, Nepal

#### Fritillaria crassicaulis

S.C. Chen, 1977

ECA206B6-1891-5CBC-8958-0C5AE3607C5B

##### Conservation status

VU

##### Distribution

China

#### Fritillaria dajinensis

S.C. Chen, 1983

4F4A47A6-3837-540A-A57A-34B5EFC5A0F9

##### Conservation status

EN

##### Distribution

China

##### Notes

Endemic to Qinghai-Tibetan Plateau

#### Fritillaria davidii

Franch., 1888

6B133295-83FA-596F-8DFE-C580D26D6557

##### Conservation status

EN

##### Distribution

China

#### Fritillaria delavayi

Franch., 1898

EE0B9C2B-025C-54EA-87ED-2CA6F63C9759

##### Conservation status

VU

##### Distribution

China, Bhutan, India

#### Fritillaria fusca

Turrill, 1943

77713CC3-A202-5336-9C6D-32C89D3153F4

##### Conservation status

EN

##### Distribution

China

##### Notes

Endemic to Qinghai-Tibetan Plateau

#### Fritillaria przewalskii

Maxim., 1882

6F1394B6-D0AD-500B-8446-169B55C7E8BF

##### Conservation status

VU

##### Distribution

China

##### Notes

Endemic to Qinghai-Tibetan Plateau

#### Fritillaria sichuanica

S.C. Chen, 1983

BEF945F6-8A13-5A6D-BBAF-434D590F61C8

##### Conservation status

VU

##### Distribution

China

##### Notes

Endemic to Qinghai-Tibetan Plateau

#### Fritillaria sinica

S.C. Chen, 1981

0E27ACFB-850F-564E-A8E0-15C40BD633B1

##### Conservation status

VU

##### Distribution

China

##### Notes

Endemic to Qinghai-Tibetan Plateau

#### Fritillaria unibracteata

Hsiao & K.C. Hsia, 1977

8522DFB7-031A-559C-BA26-A6262F18161A

##### Conservation status

EN

##### Distribution

China

##### Notes

Endemic to Qinghai-Tibetan Plateau

#### Fritillaria unibracteata var. longinectarea

S.Y. Tang & C.H. Yueh, 1991

10330C40-3E04-509F-97A2-111BE4DA6F53

##### Conservation status

CR

##### Distribution

China

##### Notes

Endemic to Qinghai-Tibetan Plateau

#### Fritillaria walujewii

Regel, 1879

FD27FD97-5D6C-5EA1-B675-51732D14FAD9

##### Conservation status

EN

##### Distribution

China, Kazakhstan

#### Lilium fargesii

Franch., 1892

AE191861-91F6-598E-AB03-3D2975AF814F

##### Conservation status

NT

##### Distribution

China

#### Lilium papilliferum

Franch., 1892

6106740F-BB09-5CAE-BAC1-377ABF59506D

##### Conservation status

NT

##### Distribution

China

#### Tulipa dasystemon

Regel, 1879

8EBD4769-BAF5-5ED2-8EC5-F187B3E8CBAD

##### Conservation status

LC

##### Distribution

China, Kazakhstan, Kyrgyzstan, Tajikistan, Uzbekistan

#### Anoectochilus roxburghii

Lindl., 1832

28FC2222-597C-5631-8781-48478AEB248B

##### Conservation status

EN

##### Distribution

China, Japan, Thailand, Laos, Vietnam, India, Bhutan, Nepal, Bangladesh

#### Anoectochilus medogensis

H. Z. Tian & Yue Jin, 2021

A02CBA8F-9642-519B-BCD2-D060D246666D

##### Conservation status

DD

##### Distribution

China

#### Bletilla striata

Rchb.f., 1878

BB725CA1-9208-53AC-94E1-95B305F87BBD

##### Conservation status

EN

##### Distribution

China, Korea, Japan

#### Calanthe dulongensis

H. Li, R. Li & Z.L. Dao, 2003

2F047B2E-CB3E-5FDE-9B8C-49B33C8755C8

##### Conservation status

CR

##### Distribution

China

##### Notes

Endemic to Qinghai-Tibetan Plateau

#### Changnienia amoena

S.S. Chien, 1935

3BC78E2C-9239-5E20-9A69-FF8CE7373E8E

##### Conservation status

EN

##### Distribution

China

#### Corybas taliensis

Tang & F.T. Wang, 1951

CD6C72AC-BA13-5628-9B7C-2B4102F99C5E

##### Conservation status

EN

##### Distribution

China

#### Cremastra appendiculata

(D. Don) Makino, 1904

2EA3C8FD-929C-534D-9F3D-90807EE34653

##### Conservation status

NT

##### Distribution

China, Nepal, Bhutan, India, Vietnam, Thailand, Japan

#### Cymbidium concinnum

Z.J. Liu & S.C. Chen, 2006

9A825F36-FD9F-5545-8009-A3A9351E9E24

##### Conservation status

DD

##### Distribution

China

#### Cymbidium cyperifolium

Wall. ex Lindl., 1833

AD85B905-4BEE-5C25-84B0-6F10857DBD08

##### Conservation status

VU

##### Distribution

China, Nepal, Bhutan, India, Myanmar, Thailand, Vietnam, Cambodia, Philippines

#### Cymbidium eburneum

Lindl., 1847

2581BE21-0024-5416-AA26-E583351118BB

##### Conservation status

EN

##### Distribution

China, Nepal, India, Myanmar

#### Cymbidium elegans

Lindl., 1833

DEF0DBE6-D592-5AD4-9B5D-6B6B3F276ADE

##### Conservation status

EN

##### Distribution

China, Nepal, Bhutan, India, Myanmar

#### Cymbidium ensifolium

(L.) Sw., 1799

83F3D4E8-C1EB-5961-BF49-10476AE462BA

##### Conservation status

VU

##### Distribution

China, Japan, Southeast and South Asia

#### Cymbidium erythraeum

Lindl., 1858

E324EFB3-502B-5628-848C-1D93F73188A4

##### Conservation status

VU

##### Distribution

China, Nepal, Bhutan, India, Myanmar

#### Cymbidium faberi

Rolfe, 1896

A327E355-F071-5F53-AB47-1003085EC8A0

##### Conservation status

EN

##### Distribution

China, Nepal, India

#### Cymbidium floribundum

Lindl., 1833

F5EAC3E3-4E88-541D-8FC9-C3B51EC83190

##### Conservation status

VU

##### Distribution

China, Vietnam

#### Cymbidium goeringii

Rchb.f., 1852

B7DD69E1-1B13-5D0F-B7D5-FC0673AB8E90

##### Conservation status

VU

##### Distribution

China, Japan, Korea

#### Cymbidium hookerianum

Rchb.f., 1866

858FB7C1-F14B-50AD-89E2-761130526E20

##### Conservation status

EN

##### Distribution

China, Nepal, Bhutan, India

#### Cymbidium iridioides

D. Don, 1825

75F56BED-FB52-5A39-9F48-741EEF629BB4

##### Conservation status

VU

##### Distribution

China, Nepal, Bhutan, India, Myanmar

#### Cymbidium kanran

Makino, 1902

FCD0327F-6013-5879-8213-3775CCD8B18B

##### Conservation status

VU

##### Distribution

China, Japan, Korea

#### Cymbidium motuoense

W. Q. Hu, Q. H. Zhang & Z. J. Liu, 2021

AC86ED34-610B-5F93-869E-D1A877AC1B2F

##### Conservation status

DD

##### Distribution

China

#### Cymbidium nanulum

Y.S. Wu & S.C. Chen, 1991

61383BEB-CDCE-5E73-8517-1A870BB48B9C

##### Conservation status

EN

##### Distribution

China

#### Cymbidium × nujiangense

X. P. Zhou, S. P. Lei & Z. J. Liu, 2007

5C936FA9-6979-540F-B2CE-CB9C055E1528

##### Conservation status

DD

##### Distribution

China

#### Cymbidium serratum

Schltr., 1919

B69A1208-4DB1-5612-BB8E-EE9A82D8D7CC

##### Conservation status

NT

##### Distribution

China

#### Cymbidium sichuanicum

Z.J. Liu & S.C. Chen, 2006

6A016AA5-6E02-5C01-BB5C-BAD1FFBE1AA9

##### Conservation status

NT

##### Distribution

China

#### Cymbidium sinense

Willd., 1805

DA794C4F-ED2A-56E5-AA12-E3C0886803FC

##### Conservation status

VU

##### Distribution

China, India, Myanmar, Vietnam, Thailand, Japan

#### Cymbidium tracyanum

L. Castle, 1890

122E3386-EAB9-5C67-8CD5-0B2FF7F193F9

##### Conservation status

LC

##### Distribution

China, Myanmar, Thailand

#### Cymbidium tortisepalum

Fukuy., 1934

DC4FB2E1-2F88-5427-90BD-7D2E71B06A2F

##### Conservation status

VU

##### Distribution

China

#### Cypripedium bardolphianum

W.W. Smith & R.J. Farrer, 1916

88A04C2A-434C-5C8D-BC10-0E100C7720A6

##### Conservation status

VU

##### Distribution

China

##### Notes

Endemic to Qinghai-Tibetan Plateau

#### Cypripedium calcicola

Schltr., 1924

DDD7A8AE-D159-574E-B1C6-1ED6ED90F348

##### Conservation status

EN

##### Distribution

China

#### Cypripedium cordigerum

D. Don, 1825

DF33A670-BF92-5220-B931-9962ED63526B

##### Conservation status

VU

##### Distribution

China, Nepal, Bhutan, India, Pakistan

#### Cypripedium debile

Rchb.f., 1874

3226C549-0522-50F1-8C7B-97E8D0F4650B

##### Conservation status

VU

##### Distribution

China, Japan

#### Cypripedium elegans

Rchb.f., 1886

4614F91A-7A1B-5ED6-A221-1E46630EB0F2

##### Conservation status

EN

##### Distribution

China, Nepal, Bhutan, India

#### Cypripedium fargesii

Franch., 1894

72046058-BAA7-52DE-9241-4B3B5E74DA7D

##### Conservation status

EN

##### Distribution

China

#### Cypripedium farreri

W.W. Sm., 1916

23EF27DD-1C0C-50E2-ADF0-C923F93A7F49

##### Conservation status

EN

##### Distribution

China

##### Notes

Endemic to Qinghai-Tibetan Plateau

#### Cypripedium fasciolatum

Franch., 1894

B9604974-415B-51BD-A18A-6A4650DAF4B5

##### Conservation status

EN

##### Distribution

China

#### Cypripedium flavum

P.F. Hunt & Summerh., 1966

CE1A7FA7-73EC-5226-BA63-3FC9672ABF82

##### Conservation status

VU

##### Distribution

China

#### Cypripedium forrestii

P.J. Cribb, 1992

29441017-054D-5803-969A-5763F9C0D681

##### Conservation status

CR

##### Distribution

China

#### Cypripedium franchetii

Rolfe, 1912

6B738A33-F238-54AD-BAD2-462CC01E6A1E

##### Conservation status

EN

##### Distribution

China

#### Cypripedium guttatum

Sw., 1800

43CEF4EE-14C1-5442-A59E-D52CF2CFC8B1

##### Conservation status

LC

##### Distribution

China, Bhutan, Korea, Russia, Europe, North America

#### Cypripedium henryi

Rolfe, 1892

396CB5F4-DC78-59BA-BB01-87DA4D85EB71

##### Conservation status

VU

##### Distribution

China

#### Cypripedium himalaicum

Rolfe ex Hemsl., 1892

1436D17A-EF95-5BD9-94E1-22AE78585E4B

##### Conservation status

EN

##### Distribution

China, Nepal, Bhutan, India

#### Cypripedium japonicum

Thunb., 1784

798708B9-AABF-59E3-84EC-E53FD1F1275A

##### Conservation status

EN

##### Distribution

China, Japan

#### Cypripedium lichiangense

S.C. Chen & P.J. Cribb, 1994

7FA203C3-836F-55E0-941E-B389C8DE8507

##### Conservation status

EN

##### Distribution

China

##### Notes

Endemic to Qinghai-Tibetan Plateau

#### Cypripedium ludlowii

P.J. Cribb, 1997

F6AEF75A-B036-535D-9CAF-D1F619787CD2

##### Conservation status

EN

##### Distribution

China

##### Notes

Endemic to Qinghai-Tibetan Plateau

#### Cypripedium macranthos

Sw., 1800

C45929FB-3CA8-543B-8A14-AB24CEA0D0EE

##### Conservation status

LC

##### Distribution

China, Japan, Korea, Russia

#### Cypripedium margaritaceum

Franch., 1888

52483670-271A-518E-8ED3-5EA963E545E9

##### Conservation status

EN

##### Distribution

China

##### Notes

Endemic to Qinghai-Tibetan Plateau

#### Cypripedium micranthum

Franchet, 1894

B9BCB1C4-9B90-52FF-B456-7EB939AF0231

##### Conservation status

EN

##### Distribution

China

#### Cypripedium palangshanense

Tang & F. T. Wang, 1936

A746198D-43B0-5705-9665-366ECB7345AC

##### Conservation status

EN

##### Distribution

China

#### Cypripedium plectrochilum

Franch., 1885

6FDA349D-666E-5EF1-97C8-24D9723DDE5E

##### Conservation status

NT

##### Distribution

China, Myanmar

#### Cypripedium shanxiense

S.C. Chen, 1983

391078ED-31D2-5481-96A8-E63975EA3159

##### Conservation status

NT

##### Distribution

China, Japan, Russia

#### Cypripedium sichuanense

Perner, 2002

23748EAA-0282-542C-841E-C9E2580690DD

##### Conservation status

EN

##### Distribution

China

##### Notes

Endemic to Qinghai-Tibetan Plateau

#### Cypripedium subtropicum

S.C. Chen & K.Y. Lang, 1986

BD816D81-ABE1-5EB0-B9EB-37AABF7D6BC6

##### Conservation status

EN

##### Distribution

China

##### Notes

Endemic to Qinghai-Tibetan Plateau

#### Cypripedium taibaiense

G.H. Zhu & S.C. Chen, 1999

229665F7-9484-532F-8066-ECAA8591BAD9

##### Conservation status

CR

##### Distribution

China

#### Cypripedium tibeticum

King ex Rolfe, 1892

A9CC7C67-6030-51BC-9166-5009A7DD7CB3

##### Conservation status

LC

##### Distribution

China, India, Bhutan

#### Cypripedium wardii

Rolfe, 1913

3AF33F13-A03A-5B34-8BF9-A80FFE2D3304

##### Conservation status

EN

##### Distribution

China

##### Notes

Endemic to Qinghai-Tibetan Plateau

#### Cypripedium yunnanense

Franch., 1894

A5D79A42-CE12-5AEF-B416-07BC97E2CF18

##### Conservation status

EN

##### Distribution

China

##### Notes

Endemic to Qinghai-Tibetan Plateau

#### Dendrobium aphyllum

C.E.C. Fisch., 1928

29CD410E-3B7D-51E0-8C7B-B113C294F28F

##### Conservation status

LC

##### Distribution

China, India, Burma, Laos, Vietnam

#### Dendrobium capillipes

Rchb.f., 1867

5FA0E986-3F07-56B6-99D8-AF73EDA91797

##### Conservation status

EN

##### Distribution

China, India, Myanmar, Thailand, Laos, Vietnam

#### Dendrobium cariniferum

Rchb.f., 1869

4937EDE9-6A30-57FB-8CF6-667A8195F733

##### Conservation status

EN

##### Distribution

China, India, Myanmar, Thailand, Laos, Vietnam

#### Dendrobium catenatum

Lindl., 1830

76F7195B-AB92-52D9-AF49-DBBD3D79A3ED

##### Conservation status

CR

##### Distribution

China, Japan

#### Dendrobium chryseum

Rolfe,1888

38937B7C-2E40-58ED-B96E-89D2F0E45F7A

##### Conservation status

EN

##### Distribution

China

#### Dendrobium chrysanthum

Wall., 1828

0BB95DC6-FAFB-5EEE-9CBD-83BA5AD48843

##### Conservation status

VU

##### Distribution

China, Nepal, Bhutan, India, Myanmar, Thailand, Laos, Vietnam

#### Dendrobium densiflorum

Lindl. ex Wall., 1830

F060A93E-DEA4-578F-A1BD-789E521F4F8C

##### Conservation status

VU

##### Distribution

China, Nepal, Bhutan, India, Myanmar, Thailand

#### Dendrobium denneanum

Kerr, 1933

B673CA66-4AC9-552D-A5F4-F296E46726BE

##### Conservation status

VU

##### Distribution

China, India, Nepal, Bhutan, Myanmar, Thailand, Laos, Vietnam

#### Dendrobium devonianum

Paxton, 1840

4DF773E6-CAE3-5BB8-B694-BADE9F74AC8A

##### Conservation status

EN

##### Distribution

China, Bhutan, India, Myanmar, Thailand, Vietnam

#### Dendrobium fimbriatum

Dalzell, 1852

3A5A6B97-F768-5DE2-849E-B0E48DBE434C

##### Conservation status

VU

##### Distribution

China, Bhutan, India, Myanmar, Nepal, Thailand, Vietnam

#### Dendrobium hancockii

Rolfe, 1903

394DDD4F-4A21-5148-A9D9-100BB7894120

##### Conservation status

EN

##### Distribution

China, Korea,Vietnam, Myanmar

#### Dendrobium heterocarpum

Wall. ex Lindl., 1830

AD92481E-FBCA-5946-A9B5-871A4D0A6BDB

##### Conservation status

VU

##### Distribution

China, Sri Lanka, India, Nepal, Bhutan, Myanmar, Thailand, Laos, Vietnam, Philippines, Malaysia, Indonesia

#### Dendrobium hookerianum

Lindl., 1858

EAB72EF2-934A-5EAF-ADD7-38D1E971C4AF

##### Conservation status

VU

##### Distribution

China, India

#### Dendrobium kwangtungense

Tso, 1933

D626669C-3A05-53DF-8AD5-7CB11F7B4546

##### Conservation status

DD

##### Distribution

China

#### Dendrobium linawianum

Rchb.f., 1861

84EF7E2C-3D4C-5698-ACB4-B9FE761ACC2D

##### Conservation status

EN

##### Distribution

China

#### Dendrobium lituiflorum

Lindl., 1856

D281DD67-A853-5AA1-89A8-8BDB9A83C34A

##### Conservation status

CR

##### Distribution

China, India, Myanmar, Thailand, Laos

#### Dendrobium longicornu

Lindl., 1829

1D337597-3060-530B-9A5A-28369A06A045

##### Conservation status

EN

##### Distribution

China, Nepal, Sikkim, Bhutan, India, Vietnam

#### Dendrobium moniliforme

(L.) Sw., 1799

2968B4D3-A7FB-5355-9F2F-AE6716DC4E05

##### Conservation status

DD

##### Distribution

China, India, Korea, Japan

#### Dendrobium monticola

P.F. Hunt & Summerh., 1961

2F968D61-65EC-56A6-B8B6-E053DABCABFF

##### Conservation status

VU

##### Distribution

China, India, Nepal, Thailand

#### Dendrobium nobile

Lindl., 1830

665DBA8E-1244-596A-B70D-725CC9C6AE32

##### Conservation status

VU

##### Distribution

China, India, Nepal, Bhutan, Myanmar, Thailand, Laos, Vietnam

#### Dendrobium officinale

Kimura & Migo, 1936

E763A9B3-A353-5F48-983C-3174C9368D9C

##### Conservation status

CR

##### Distribution

China, Japan

#### Dendrobium salaccense

(Blume) Lindl., 1830

C9285EEB-4ED3-5827-82EC-9D0FB8D12CD8

##### Conservation status

VU

##### Distribution

China, Bhutan, India, Indonesia, Laos, Malaysia, Myanmar, Sri Lanka, Thailand, Vietnam

#### Dendrobium stuposum

Lindl., 1838

A013CA5F-3A64-5485-9F71-8FCD84ED867E

##### Conservation status

VU

##### Distribution

China, Bhutan, India, Indonesia, Malaysia, Myanmar, Philippines, Thailand

#### Dendrobium williamsonii

Day & Rchb.f., 1869

73AB25FF-9F33-5D88-B7C4-84D3B04651BC

##### Conservation status

EN

##### Distribution

China, India, Burma, Vietnam

#### Gastrodia angusta

S. Chow & S.C. Chen， 1983

B49372F0-A133-5221-8179-15CCAABFD2D9

##### Conservation status

EN

##### Distribution

China

##### Notes

Endemic to Qinghai-Tibetan Plateau

#### Gastrodia elata

Blume, 1856

233E09E9-7E5A-5633-B364-0E4774EE2647

##### Conservation status

VU

##### Distribution

China, Bhutan, India, Japan, Korea, Nepal, Russia

#### Gymnadenia conopsea

(L.) R.Br., 1813

05D989E2-7014-5995-9874-97E77FE7797A

##### Conservation status

DD

##### Distribution

China, Japan, Korea, Russia, Europe

#### Gymnadenia orchidis

Lindl., 1835

C6DAEAF5-E4C7-5970-9CB2-32BBCCF1F595

##### Conservation status

VU

##### Distribution

China, Bhutan, India, Pakistan

#### Paphiopedilum armeniacum

S.C. Chen & F.Y. Liu, 1982

119EEB8A-8558-559A-B179-358B259CE79A

##### Conservation status

EN

##### Distribution

China, Myanmar

#### Paphiopedilum insigne

(Wall. ex Lindl.) Pfitzer, 1888

5C4A45BF-F0F7-5EEE-A137-786355F3E972

##### Conservation status

CR

##### Distribution

China, India

#### Paphiopedilum spicerianum

(Rchb.f.) Pfitzer, 1888

B9A4FFFC-C117-5546-995C-CA814A30C2A6

##### Conservation status

CR

##### Distribution

China, India, Vietnam

#### Paphiopedilum tigrinum

Koop. & N. Haseg., 1990

EDA98EDD-EE7D-5191-9C20-0CF17F254FA1

##### Conservation status

EN

##### Distribution

China, China

#### Paphiopedilum venustum

(Wall. ex Sims) Pfitzer, 1888

252D2150-15CC-5F7B-9B3C-33F4D0AE8C94

##### Conservation status

EN

##### Distribution

China, Nepal, Bhutan, Sikkim, India, Bangladesh

#### Paphiopedilum wardii

Summerh., 1932

7C5C3D3E-C5F2-5AF0-B2F5-FD21D94B8BB1

##### Conservation status

EN

##### Distribution

China, Myanmar

#### Phalaenopsis wilsonii

Rolfe, 1909

9CE753AB-C97E-56C5-9482-CBB6DF337F5E

##### Conservation status

VU

##### Distribution

China, India, Vietnam

#### Pleione arunachalensis

Hareesh, Kumar & M. Sabu, 2017

97D5518A-1345-5112-8A90-E708002308CE

##### Conservation status

CR

##### Distribution

China, China

#### Pleione bulbocodioides

(Franch.) Rolfe, 1903

A17DBF11-7B6A-5F85-8CEE-861A112A1A2C

##### Conservation status

DD

##### Distribution

China, Endemic to China

#### Pleione formosana

Hayata, 1911

E8F4B25C-79CE-5205-8D5B-1D8389BFAAB9

##### Conservation status

VU

##### Distribution

China, Endemic to China

#### Pleione forrestii

Schltr., 1912

8FB1C684-F180-5B18-81C2-989ECB0FF21C

##### Conservation status

EN

##### Distribution

China, Endemic to China

#### Pleione grandiflora

(Rolfe) Rolfe, 1903

00EAE830-01EF-5184-A691-DF70EE6847A6

##### Conservation status

CR

##### Distribution

China, Vietnam

#### Pleione hookeriana

(Lindl.) Rollisson, 1875

A62EC86D-09D9-5A42-A178-B45805852D58

##### Conservation status

VU

##### Distribution

China, Nepal, Bhutan, India, Myanmar, Laos,Thailand

#### Pleione kaatiae

P.H. Peeters, 2003

44ACD94D-3B65-5E5E-9688-5F4B56FD3DC3

##### Conservation status

DD

##### Distribution

China, China

#### Pleione limprichtii

Schltr., 1922

92B63679-E0D3-5C98-AD6E-4F9CB3E00413

##### Conservation status

VU

##### Distribution

China, Myanmar

#### Pleione praecox

D. Don, 1825

5A967E04-C6FF-5E3B-B6C6-49649DE9B7B7

##### Conservation status

VU

##### Distribution

China, Bangladesh, Bhutan, India, Laos, Myanmar, Nepal, Thailand, Vietnam

#### Pleione saxicola

Tang & F.T. Wang ex S.C. Chen, 1987

8A7ABDE9-231A-5D77-8746-EFF909F1E51A

##### Conservation status

EN

##### Distribution

China, Bhutan

#### Pleione scopulorum

W.W. Sm., 1921

0382D857-AFD6-57AF-B945-B73EB6FD94C1

##### Conservation status

VU

##### Distribution

China, India, Myanmar

#### Pleione yunnanensis

(Rolfe) Rolfe, 1903

9F9CD5D6-B727-5F2C-90C2-B021871EF765

##### Conservation status

VU

##### Distribution

China, Myanmar

#### Iris narcissiflora

Diels, 1924

2EFD04EF-FE55-5B1B-8B97-F25D984C3C36

##### Conservation status

VU

##### Distribution

China

##### Notes

Endemic to Qinghai-Tibetan Plateau

#### Caryota obtusa

Griff., 1845

11E831FE-101C-5E2F-AB44-68E19A3028D9

##### Conservation status

VU

##### Distribution

China, India, Laos, Myanmar, Thailand, Vietnam

#### Trachycarpus nanus

Becc., 1910

3C8FA27C-541F-5DE3-B17E-DA6FB712722E

##### Conservation status

EN

##### Distribution

China

#### Achnatherum breviaristatum

Keng & P.C. Kuo, 1976

EB58F4D8-D67D-5ABA-B716-B4814D03A624

##### Conservation status

VU

##### Distribution

China

#### Agropyron mongolicum

Keng, 1938

58EBE1D6-83E9-5D60-81E9-8831BFBA1866

##### Conservation status

LC

##### Distribution

China

#### Aristida triseta

Keng, 1941

5F915A67-D22A-559F-99BE-139E76EBBC54

##### Conservation status

LC

##### Distribution

China

##### Notes

Endemic to Qinghai-Tibetan Plateau

#### Elymus alashanicus

(Keng) S.L. Chen, 1994

7DCDEB86-ECE4-5B9E-94AE-906C2F5276B5

##### Conservation status

LC

##### Distribution

China

#### Elymus atratus

(Nevski) Hand.-Mazz., 1936

80BEC8A6-C8CF-556D-AC05-88A4C0F4FB54

##### Conservation status

LC

##### Distribution

China

##### Notes

Endemic to Qinghai-Tibetan Plateau

#### Elymus brevipes

(Keng) S.L. Chen, 2006

BA6051C0-C795-532C-A20F-BD55CD2B888A

##### Conservation status

LC

##### Distribution

China

##### Notes

Endemic to Qinghai-Tibetan Plateau

#### Elymus purpuraristatus

C.P. Wang & H.L. Yang, 1984

263E4959-4EA4-5F2E-AE61-CFA2E7C2748C

##### Conservation status

LC

##### Distribution

China

#### Elymus sinosubmuticus

S.L. Chen, 2006

57DE6885-E791-549C-B821-5920C5F043CD

##### Conservation status

VU

##### Distribution

China

#### Gaoligongshania megalothyrsa

(Hand.-Mazz.) D.Z. Li, Hsueh & N.H. Xia, 1995

23618713-8147-5E18-9B76-57335AD1709E

##### Conservation status

NT

##### Distribution

China

#### Kengyilia kokonorica

(Keng) J.L. Yang, C. Yen & B.R. Baum, 1992

0859109B-164C-51C6-875A-71C08EBF1130

##### Conservation status

LC

##### Distribution

China

##### Notes

Endemic to Qinghai-Tibetan Plateau

#### Orinus kokonorica

(K.S. Hao) Keng, 1957

AAE46E8B-9BB4-5F7D-B2FF-1B029958E33C

##### Conservation status

LC

##### Distribution

China

##### Notes

Endemic to Qinghai-Tibetan Plateau

#### Sinochasea trigyna

Keng, 1958

6C4130F0-238D-5AB4-90AE-6B7521D88B11

##### Conservation status

VU

##### Distribution

China

#### Hordeum innermongolicum

P.C. Kuo & L.B. Cai, 1987

1E202C84-FD23-5165-BBB2-79E4DB0FE674

##### Conservation status

VU

##### Distribution

China

#### Meconopsis barbiseta

C.Y. Wu & H. Chuang ex L.H. Zhou, 1979

A1FB0704-652F-5B4D-9008-28426DA6FD06

##### Conservation status

EN

##### Distribution

China

##### Notes

Endemic to Qinghai-Tibetan Plateau

#### Meconopsis punicea

Maxim., 1889

CAD7FA84-217D-5CDA-9D9D-76958191B497

##### Conservation status

LC

##### Distribution

China

##### Notes

Endemic to Qinghai-Tibetan Plateau

#### Meconopsis torquata

Prain, 1906

77DFFF65-3E23-5D79-A16B-C98BB7F951DE

##### Conservation status

NT

##### Distribution

China

##### Notes

Endemic to Qinghai-Tibetan Plateau

#### Corydalis saxicola

G.S. Bunting, 1966

E05EF355-E784-58C1-BAB3-06B46C1291DA

##### Conservation status

LC

##### Distribution

China

#### Dysosma aurantiocaulis

(Hand.-Mazz.) Hu, 1937

27D0B15F-E4A2-5AEF-82CC-DFB0A279F819

##### Conservation status

VU

##### Distribution

China, Myanmar

#### Dysosma delavayi

(Franch.) Hu, 1937

EE4D4CED-63C5-524D-869E-59C9D2B8BF0C

##### Conservation status

DD

##### Distribution

China

#### Dysosma tsayuensis

T.S. Ying, 1979

EDF650E1-836C-50BC-AF9C-E08572B8BE8B

##### Conservation status

VU

##### Distribution

China

##### Notes

Endemic to Qinghai-Tibetan Plateau

#### Dysosma versipellis

(Hance) M. Cheng ex T.S. Ying, 1979

FBB3E531-C566-5BFE-AFA7-1385F891B663

##### Conservation status

VU

##### Distribution

China

#### Sinopodophyllum hexandrum

(Royle) T.S. Ying, 1985

5B17580E-34AF-5175-BD1D-343EB8DA0C9C

##### Conservation status

LC

##### Distribution

China, Afghanistan, Bhutan, India, Nepal, Pakistan

#### Kingdonia uniflora

Balf. f. & W.W. Sm., 1914

DB4CF17F-29DD-557B-87E3-3C2985405D58

##### Conservation status

VU

##### Distribution

China

#### Coptis chinensis

Franch., 1897

EA7C64AD-E854-5831-982B-A436CCD210EB

##### Conservation status

VU

##### Distribution

China

#### Coptis teeta

Wall., 1842

A7BFB6C5-CA20-5EE6-A478-B581BC01B573

##### Conservation status

EN

##### Distribution

China

##### Notes

Endemic to Qinghai-Tibetan Plateau

#### Nelumbo nucifera

Gaertn., 1788

4985E714-DD5D-58D2-AABC-3A2E076CA114

##### Conservation status

DD

##### Distribution

China, Bhutan, India, Indonesia, Japan, Korea, Malaysia, Myanmar, Nepal, New Guinea, Pakistan, Philippines, Russia, Sri Lanka, Thailand, Vietnam, Australia

#### Tetracentron sinense

Oliv., 1889

0CF83697-9F7B-5616-BAFD-F3D16508EDA5

##### Conservation status

LC

##### Distribution

China, Bhutan, India, Myanmar, Nepal, Vietnam

#### Paeonia delavayi

Franch., 1887

056754F1-0F5A-5903-8893-4A8E8CC85285

##### Conservation status

LC

##### Distribution

China

##### Notes

Endemic to Qinghai-Tibetan Plateau

#### Paeonia decomposita

Hand.-Mazz., 1939

2E8051A9-03B9-5C6E-963A-12849514199D

##### Conservation status

EN

##### Distribution

China

##### Notes

Endemic to Qinghai-Tibetan Plateau

#### Paeonia ludlowii

(Stern & G.Taylor) D.Y. Hong, 1997

8B021DC3-9C34-557E-A4C4-5E2808DB59D7

##### Conservation status

EN

##### Distribution

China

##### Notes

Endemic to Qinghai-Tibetan Plateau

#### Paeonia rockii

(S.G. Haw & Lauener) T. Hong & J.J. Li, 1992

B5182BFD-1A34-525E-840F-FC52A406076D

##### Conservation status

VU

##### Distribution

China

#### Paeonia rockii linyanshanii

T. Hong & G.L. Osti, 1994

90264D31-47AF-5D68-A698-727F2E70D5C8

##### Conservation status

DD

##### Distribution

China

#### Paeonia rotundiloba

D.Y. Hong, 2011

5F37A77D-C0F6-5CCC-90E6-2BBF2EAD9F8C

##### Conservation status

EN

##### Distribution

China

#### Paeonia sterniana

H.R. Fletcher, 1959

CC79DB92-61FA-5273-ABE2-244CF0F11010

##### Conservation status

LC

##### Distribution

China

#### Cercidiphyllum japonicum

Siebold & Zucc., 1846

F9048ADE-AF5D-5B40-859F-71D5BCA59CCA

##### Conservation status

LC

##### Distribution

China, Japan

#### Rhodiola crenulata

(Hook.f. & Thomson) H. Ohba, 1976

72E4DDFD-E3CF-5943-82C3-F6BE2841F001

##### Conservation status

EN

##### Distribution

China, Bhutan, Nepal, India

#### Rhodiola fastigiata

(Hook.f. & Thomson) Fu, 1965

9ADA9F3F-B63D-527A-9E6D-DE052C276A3A

##### Conservation status

LC

##### Distribution

China, Bhutan, India, Kashmir, Nepal

#### Rhodiola himalensis

(D.Don) Fu, 1965

DA2488B8-4FDC-5EA9-9AF7-D32986D49F93

##### Conservation status

LC

##### Distribution

China, Bhutan, Nepal, India

#### Rhodiola quadrifida

Fisch. & C.A. Mey., 1841

0362CAAA-84C5-547D-BA27-297DFCCE0212

##### Conservation status

LC

##### Distribution

China, Kazakhstan, Mongolia, Russia

#### Rhodiola rosea

L., 1753

8A850046-B778-56AE-AD52-162314D5C61D

##### Conservation status

VU

##### Distribution

China, Japan, Kazakhstan, Korea, Mongolia, Russia; Europe, North America

#### Rhodiola sacra

(Raym.-Hamet) Fu, 1965

E6781ADC-0BB1-5D09-B480-7B3FCC6449B4

##### Conservation status

VU

##### Distribution

China

#### Rhodiola tangutica

(Maxim.) S.H. Fu, 1986

B9A3E290-BBAE-5CF7-9282-BBDB0F9C596F

##### Conservation status

VU

##### Distribution

China

##### Notes

Endemic to Qinghai-Tibetan Plateau

#### Rhodiola wallichiana

(Hook.) Fu, 1965

41E7B0F9-21D0-55DF-A58C-176E0270AD7D

##### Conservation status

LC

##### Distribution

China, Bhutan, India, Myanmar, Nepal

#### Rhodiola yunnanensis

(Franch.) Fu, 1965

7F48E688-8385-5F65-98F6-15DB562B05A9

##### Conservation status

LC

##### Distribution

China

#### Cynomorium songaricum

Rupr., 1869

501D23AB-99D8-5B6F-A1CC-5BF4AD8160FF

##### Conservation status

VU

##### Distribution

China, Afghanistan, Mongolia, Iran

#### Glycine soja

Siebold & Zucc., 1845

9CD60931-5019-5166-B16D-9E7C0621BE38

##### Conservation status

LC

##### Distribution

China, Afghanistan, Japan, Korea, Russia

#### Glycyrrhiza inflata

Batalin, 1891

DE9DD9A4-16DD-5886-88FA-7F7837A3D431

##### Conservation status

LC

##### Distribution

China, Kazakhstan, Kyrgyzstan, Mongolia, Tajikistan, Turkmenistan, Uzbekistan

#### Glycyrrhiza uralensis

Fisch. ex DC., 1825

41E18EFC-BC08-52CB-B083-B8627E65FF44

##### Conservation status

LC

##### Distribution

China, Mongolia, Russia

#### Ormosia henryi

Prain, 1900

2CBA13D2-654E-57BA-AFD3-ED4335795CCE

##### Conservation status

VU

##### Distribution

China, Vietnam,Thailand

#### Ormosia hosiei

Hemsl. & E.H. Wilson, 1906

2BCC7310-0333-56FA-ACDC-93DC40F10CD8

##### Conservation status

EN

##### Distribution

China

#### Salweenia bouffordiana

H. Sun, Zhi M. Li & J.P. Yue, 2011

BAEE1BA4-9848-510C-A491-8DDD609B1AFB

##### Conservation status

EN

##### Distribution

China

##### Notes

Endemic to Qinghai-Tibetan Plateau

#### Salweenia wardii

Baker f., 1935

CC75B0A2-953F-53CA-8BAC-9F20643E9867

##### Conservation status

EN

##### Distribution

China

##### Notes

Endemic to Qinghai-Tibetan Plateau

#### Malus rockii

Rehder, 1933

8EAC9EC4-1DF6-5341-A406-05B0B91AF478

##### Conservation status

LC

##### Distribution

China, Bhutan

#### Malus sieversii

M.Roem., 1847

D31D2781-5A13-5837-8A43-125FEE88B5C1

##### Conservation status

VU

##### Distribution

China, Kazakhstan, Russia

#### Malus sikkimensis

(Wenz.) Koehne ex C.K. Schneid., 1906

35281BD6-42C5-5F40-81BE-0CE0762629A3

##### Conservation status

DD

##### Distribution

China, Bhutan, India, Nepal

#### Prunus kansuensis

Rehder, 1922

C53D71A2-5382-559E-AAC9-279C9856BF7F

##### Conservation status

DD

##### Distribution

China

#### Prunus mira

Koehne, 1912

2088CB51-FC8B-5FAA-92B3-2D7761D26550

##### Conservation status

DD

##### Distribution

China

##### Notes

Endemic to Qinghai-Tibetan Plateau

#### Prunus mongolica

Maxim., 1879

1AC90916-2C27-513E-85AB-0D23C5132E14

##### Conservation status

VU

##### Distribution

China, Mongolia

#### Prunus tenella

Batsch, 1801

86B21B86-8F50-53BD-B872-281113D20CD2

##### Conservation status

EN

##### Distribution

Europe, West and Central Asia

#### Rosa chinensis var. spontanea

(Rehder & E.H. Wilson) T.T. Yu & T.C. Ku, 1985

02684F02-80B3-581B-9E61-39D503179A22

##### Conservation status

EN

##### Distribution

China

#### Rosa lucidissima

H. Lév., 1911

948EA5C6-4EDF-594B-8A2E-C17F5359338C

##### Conservation status

CR

##### Distribution

China

#### Rosa odorata var. gigantea

(Crép.) Rehder & E.H. Wilson, 1915

8548FF1B-910E-5AD8-807D-16F3EB4E4841

##### Conservation status

DD

##### Distribution

China, Myanmar, Thailand, Vietnam

#### Rosa praelucens

Bijh., 1929

D65F1D41-5F5C-54A1-BEE7-37FDBC94D2BF

##### Conservation status

CR

##### Distribution

China

#### Berchemiella wilsonii

(C.K. Schneid.) Nakai, 1923

3E8AF447-982E-59C2-8BFC-F8EBBB888408

##### Conservation status

LC

##### Distribution

China

#### Zelkova schneideriana

Hand.-Mazz., 1929

52492CC3-0B42-5731-9632-2840B5B41F0F

##### Conservation status

VU

##### Distribution

China

#### Morus macroura

Miq., 1851

DFFD969A-F339-57BB-A20A-330A20763A6A

##### Conservation status

LC

##### Distribution

China, Bhutan, Indochina, Malaysia, Myanmar, India, Thailand

#### Morus notabilis

C.K. Schneid., 1916

18165448-F585-54C6-A7BA-88BA7E666788

##### Conservation status

LC

##### Distribution

China

#### Quercus oxyphylla

Hand.-Mazz., 1929

8529B8CD-7343-5C58-8940-0005A613EF65

##### Conservation status

NT

##### Distribution

China

#### Sapria himalayana

Griffi., 1844

C4DDA2D8-E893-5C2C-BB31-00D5E10940E5

##### Conservation status

VU

##### Distribution

China, India, Myanmar, Thailand, Vietnam

#### Terminalia myriocarpa

Van Heurck & Müll. Arg., 1871

8D58A438-86FA-5C03-BE90-6812B897CD74

##### Conservation status

VU

##### Distribution

China, Bangladesh, Bhutan, India, Indonesia, Laos, Malaysia, Myanmar, Nepal, Thailand, Vietnam

#### Lagerstroemia minuticarpa

Debberm. ex P.C. Kanjilal, 1934

A08C8B90-6F0D-5E31-86B3-DD2BBDA0A672

##### Conservation status

EN

##### Distribution

China, India, Bhutan

#### Trapa incisa

Sieb. et Zucc.., 1845

C8291B12-A8FF-5274-BE98-8F3A1E87C75C

##### Conservation status

DD

##### Distribution

China, Japan, Vietnam, Thailand, Laos, Malaysia, Indonesia

#### Acer amplum catalpifolium

(Rehder) Y.S. Chen, 2008

05DEAEE1-4741-530F-A070-7C9866271D71

##### Conservation status

VU

##### Distribution

China

#### Acer pentaphyllum

Diels, 1931

6B238C56-6DE2-55BA-A3B3-61D557107928

##### Conservation status

CR

##### Distribution

China

##### Notes

Endemic to Qinghai-Tibetan Plateau

#### Eurycorymbus cavaleriei

(H. Lév.) Rehder & Hand.-Mazz., 1934

B41650CF-C8EB-5BAE-B58D-64F36A281D26

##### Conservation status

NT

##### Distribution

China

#### Handeliodendron bodinieri

(H. Lév.) Rehder, 1935

EA286958-6B9C-52B9-9EA0-1EF9D054C3A4

##### Conservation status

EN

##### Distribution

China

#### Citrus cavaleriei

H. Lév. ex Cavalerie, 1911

74F7C124-7588-5821-A7F7-1E046C5339E7

##### Conservation status

DD

##### Distribution

China

#### Phellodendron chinense

C.K. Schneid., 1907

660CF5CB-CDBB-5443-91B4-EDD5786E5FF5

##### Conservation status

LC

##### Distribution

China

#### Aglaia lawii

(Wight) C.J. Saldanha, 1976

37B9B735-DD3C-5573-B74F-1518EEB4A6B0

##### Conservation status

LC

##### Distribution

China, Indonesia, Laos, Malaysia, Thailand, Vietnam

#### Toona ciliata

M. Roem., 1846

12361DEA-5576-5657-B1B9-72FEBEBB20F8

##### Conservation status

LC

##### Distribution

China, Bangladesh, Bhutan, Cambodia, India, Indonesia, Laos, Malaysia, Myanmar, Nepal, Pakistan, Papua New Guinea, Philippines, Sri Lanka, Thailand, Vietnam, Australia

#### Paradombeya sinensis

Dunn, 1902

026E4043-4ED0-5F1F-AEFC-4913FECC59FD

##### Conservation status

EN

##### Distribution

China

#### Hopea shingkeng

(Dunn) Bor, 1941

DD824B8B-9D57-505B-9D76-0A55BED01559

##### Conservation status

EN

##### Distribution

China

#### Firmiana major

(W. W. Smith) Hand.-Mazz., 1878

73FAE8B8-61A4-5EB6-8F0D-FB2EAD7B2E0D

##### Conservation status

EN

##### Distribution

China

#### Fagopyrum dibotrys

(D.Don) H. Hara, 1966

028F7210-94DA-5662-85CA-BC233E9FD972

##### Conservation status

LC

##### Distribution

China, Bhutan, India, Kashmir, Myanmar, Nepal, Vietnam

#### Psammosilene tunicoides

W.C. Wu & C.Y. Wu, 1945

47FB279C-D834-5266-A0BA-3835A7B366E1

##### Conservation status

EN

##### Distribution

China

#### Sinojackia henryi

(Dümmer) Merr., 1937

3663590F-0A6B-5470-A507-4EBE7F21CF70

##### Conservation status

DD

##### Distribution

China

#### Baolia bracteata

H.W. Kung & G.L. Chu, 1978

756117FC-7136-52CC-A58C-C40AE26DCACA

##### Conservation status

LC

##### Distribution

China

##### Notes

Endemic to Qinghai-Tibetan Plateau

#### Davidia involucrata

Baillon, 1871

BFE86EA8-E1D6-5624-984E-EDE5867F6A50

##### Conservation status

LC

##### Distribution

China

#### Pomatosace filicula

Maxim., 1881

50F162B3-A494-53F9-B87C-8229969B1E75

##### Conservation status

LC

##### Distribution

China

##### Notes

Endemic to Qinghai-Tibetan Plateau

#### Camellia sinensis

(L.) Kuntze, 1887

5CD28C3F-CB2C-5E55-A42B-CB592AFFCAD8

##### Conservation status

DD

##### Distribution

China, India, Japan, Korea

#### Camellia sinensis var. assamica

(J.W. Mast.) Kitam., 1950

CD5C3193-F515-5EE6-83F5-E352B5C67D6C

##### Conservation status

VU

##### Distribution

China, Laos, Myanmar, Thailand, Vietnam

#### Actinidia arguta

Miq., 1867

A6FB9143-9DBD-563C-A048-34E8EADD284A

##### Conservation status

LC

##### Distribution

China, Japan, Korea

#### Actinidia chinensis

Planch., 1847

2BA356B4-D2AB-5B8C-9B66-EC1D8EB118E5

##### Conservation status

LC

##### Distribution

China

#### Rhododendron dauricum

L., 1753

D4E86258-4363-5AA9-8598-B7F6EF2A644A

##### Conservation status

LC

##### Distribution

China, Japan, Korea, Mongolia, Russia

#### Rhododendron williamsianum

Rehder & E.H. Wilson, 1913

2AE93786-FA0A-54D7-9B8A-44CA3AFE0981

##### Conservation status

EN

##### Distribution

China

#### Emmenopterys henryi

Oliv., 1889

97E81E58-9A79-595A-81A4-024289899EF5

##### Conservation status

NT

##### Distribution

China

#### Neonauclea tsaiana

S.Q. Zou, 1988

3279BDF8-200A-5E2E-9833-DE655B2623E5

##### Conservation status

EN

##### Distribution

China

#### Lomatogoniopsis alpina

T.N. Ho & S.W. Liu, 1980

6459D2FE-BB52-502C-8F1A-42B384A166FA

##### Conservation status

EN

##### Distribution

China

##### Notes

Endemic to Qinghai-Tibetan Plateau

#### Merrillanthus hainanensis

Chun & Tsiang, 1941

A78E0853-5D65-506F-9FBC-B164F2A7B4A6

##### Conservation status

EN

##### Distribution

China, Cambodia

#### Arnebia euchroma

(Royle ex Benth.) I.M. Johnst., 1924

52847350-9E42-5C11-8121-F0C92F44A47A

##### Conservation status

EN

##### Distribution

China, Afghanistan, India, Kazakhstan, Kyrgyzstan, Nepal, Pakistan, Russia, Tajikistan, Turkmenistan, Uzbekistan

#### Lycium ruthenicum

Murray, 1780

023ABD17-87D9-5B6B-B958-B0D271C10FA3

##### Conservation status

LC

##### Distribution

China, Afghanistan, Kazakhstan, Kyrgyzstan, Mongolia, Pakistan, Russia, Tajikistan, Turkmenistan, Uzbekistan, Georgia,Turkey, Azerbaijan

#### Fraxinus mandshurica

Rupr., 1857

E04F8FEC-9877-5249-9C66-C393504A7253

##### Conservation status

VU

##### Distribution

China, Japan, Korea, Russia

#### Fraxinus sogdiana

Bunge, 1852

A2266DD7-E0AC-5AAC-9143-16844E2AEEBB

##### Conservation status

LC

##### Distribution

China, Kazakhstan, Kyrgyzstan, Tajikistan, Uzbekistan

#### Neopicrorhiza scrophulariiflora

(Pennell) D.Y. Hong, 1984

873C2548-6050-5F45-8EE0-F9A2C661910E

##### Conservation status

EN

##### Distribution

China, Nepal

#### Scrophularia stylosa

P.C. Tsoong, 1994

AA9FFABF-CD7A-55AF-9DBB-1557D0411087

##### Conservation status

VU

##### Distribution

China

#### Cistanche deserticola

Ma, 1960

BDEF3A1F-7193-54E8-8177-18E94C409B22

##### Conservation status

EN

##### Distribution

China, Mongolia

#### Leucomeris decora

Kurz, 1872

5C1DE6A6-F79B-5371-AAAC-83F0A84C2D86

##### Conservation status

DD

##### Distribution

China, Vietnam, Thailand, Myanmar

#### Saussurea balangshanensis

Ya Z. Zhang & H. Sun, 2019

90D29704-4665-5352-B02A-75027F048503

##### Conservation status

DD

##### Distribution

China

##### Notes

Endemic to Qinghai-Tibetan Plateau

#### Saussurea gossipiphora

D. Don, 1821

BCBC7A06-0039-57DF-A6A3-33E55815015E

##### Conservation status

LC

##### Distribution

China, India, Pakistan, Nepal, Bhutan

#### Saussurea laniceps

Hand.-Mazz., 1937

B3BCDC4F-7332-53A4-8610-198C306FCBB3

##### Conservation status

DD

##### Distribution

China

##### Notes

Endemic to Qinghai-Tibetan Plateau

#### Saussurea medusa

Maxim., 1881

417BAF6E-07BD-53F4-9F1E-2BE34E06A08D

##### Conservation status

DD

##### Distribution

China, India, Pakistan

#### Nardostachys jatamansi

(Jones) DC., 1830

CD278B14-9F1A-5F35-A6E7-4514D81F61B9

##### Conservation status

CR

##### Distribution

China, India, Nepal, Bhutan

#### Panax bipinnatifidus

Seem.,1868

705449EF-28B9-5AD8-A976-79C107AC6102

##### Conservation status

VU

##### Distribution

China, India, Nepal, Vietnam

#### Panax bipinnatifidus var. angustifolius

(Burkill) J. Wen, 2001

276A9632-F351-559D-8B02-42E6F9877EC9

##### Conservation status

DD

##### Distribution

China, Bhutan, India, Nepal, Thailand

#### Panax pseudo-ginseng

C.A. Mey., 1842

24BD204C-AEA3-559E-9DCE-E28F13EA9A0B

##### Conservation status

LC

##### Distribution

China

##### Notes

Endemic to Qinghai-Tibetan Plateau

#### Panax stipuleanatus

Tsai & K.M. Feng, 1975

40AC911D-77B8-5468-91CC-A000CDDBA06C

##### Conservation status

EN

##### Distribution

China, Vietnam

#### Panax vietnamensis

Ha & Grushvitzky, 1985

A31B6874-68DA-581F-9A42-0D7CBCD2E8E5

##### Conservation status

DD

##### Distribution

China, Vietnam

#### Panax wangianum

S.C. Sun, 1946

53B2E855-2A3A-5BCB-9231-24593F56CC82

##### Conservation status

DD

##### Distribution

China

#### Chuanminshen violaceum

M.L. Sheh & R.H. Shan, 1980

DA163256-47C1-5E8C-A076-72B3595A948A

##### Conservation status

EN

##### Distribution

China

## Analysis

### Statistical analyses of families and genus

There were 330 species, 18 varieties and two subspecies of national key protected wild plants on the Qinghai-Tibetan Plateau, belonging to 72 families and 130 genera, including five species of Bryophyta in three families and three genera, 27 species of Lycopodiophyta in two families and three genera, 11 species of Pteridophyta in three families and six genera, 25 species of Gymnospermae in five families and 12 genera, 262 species, 18 varieties and two subspecies of Angiospermae in 59 families and 106 genera (Suppl. material 1). These accounted for 1.43%, 7.71%, 3.14%, 7.14% and 80.57% of the national key protected wild plants on the Qinghai-Tibetan Plateau, respectively.

The *List* (2021) contains approximately 1,101 species (455 species and 40 categories), including five species of Bryophyta, eight species and seven categories of Lycopodiophyta and Pteridophyta (~ 106 species), 35 species and seven categories of Gymnospermae (~ 107 species), 397 species and 26 categories of Angiospermae (~ 830 species). According to the *List* (2021), the nationally protected wild plants distributed on the Qinghai-Tibetan Plateau accounted for 31.88% of the total in the *List* (2021), amongst which Bryophyta were all distributed on the Plateau, Lycopodiophyta and Pteridophyta, Gymnospermae and Angiospermae, accounted for 35.85%, 23.36% and 34.10% of the total species in the *List* (2021), respectively.

**Figure 1.**
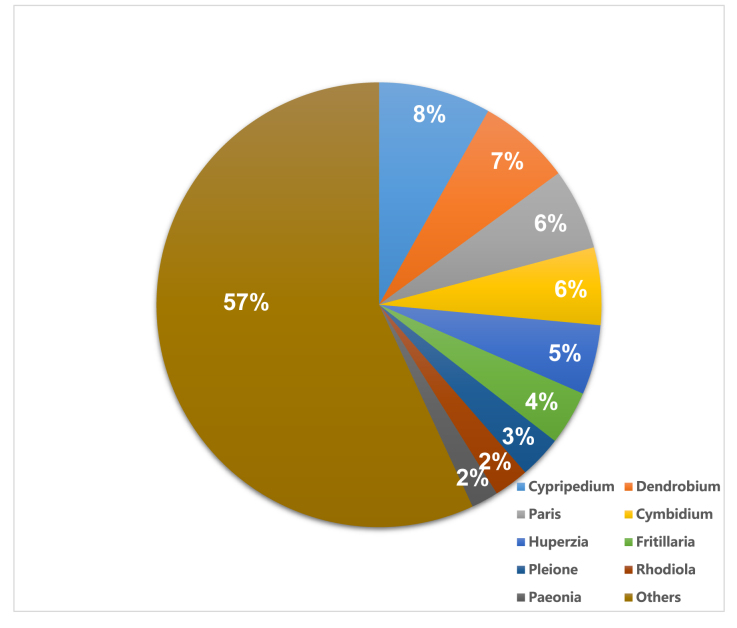
Statistics of the proportion of each family of national key protected wild plants on the Qinghai-Tibetan Plateau.

In terms of family composition, the national key protected wild plants on the Qinghai-Tibetan Plateau belonged to 72 families. Orchidaceae was the only family with over 100 species, with 103 species accounting for 29.34% of the national key protected wild plants (350 species) on the Qinghai-Tibetan Plateau. This was followed by Lycopodiaceae (24 species) and Melanthiaceae (21 species), which accounted for 6.83% and 5.98% of the national key protected wild plant species (350 species), respectively. Families with over 10 species also included Liliaceae (15 species), Poaceae (13 species), Rosaceae (11 species) and Taxaceae (10 species). A total of 33 families had only one species, accounting for 45.83% of all families (Fig. 1).

**Figure 2.**
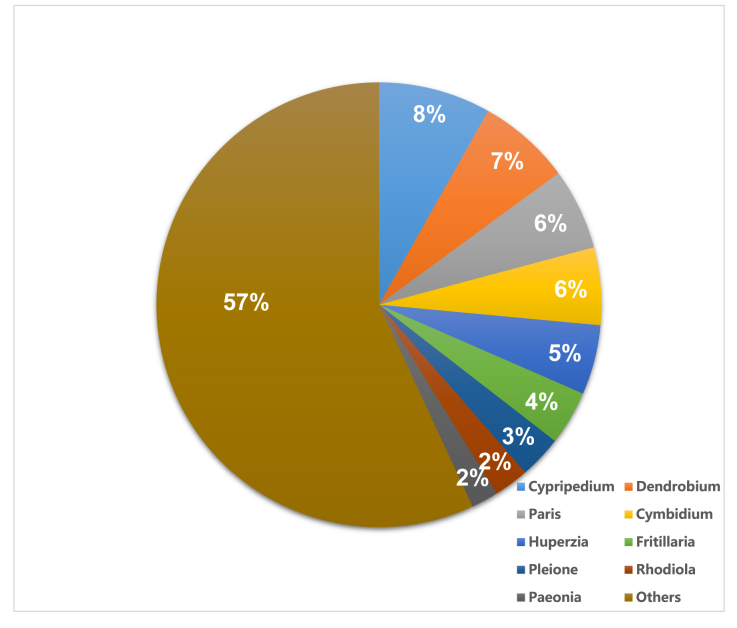
Statistics of the proportion of each genus of national key protected wild plants on the Qinghai-Tibetan Plateau.

In terms of genus composition, the national key protected wild plants on the Qinghai-Tibet Plateau belonged to 130 genera. Genera with more than 20 species included Cypripedium (29 species), Dendrobium (24 species) and Paris (21 species), accounting for 8.26%, 6.84%, and 5.98% of the total species (350 species) on the Qinghai-Tibetan Plateau, respectively. However, the genus with only one species included 77 genera, accounting for 59.23% of all the genera (Fig. 2).

### Endemic and endangered status of taxa

According to statistics, there are 14,939 species of endemic seed plants in China and 3,764 species of endemic seed plants in the Qinghai-Tibetan Plateau (Yu et al. 2018). The national key protected wild plants distributed on the Qinghai-Tibetan Plateau included 168 species endemic to China, including 16 species of Lycopodiophyta, three species of Pteridophyta, 14 species of Gymnospermae and 135 species of Angiospermae. The species of seed plants (Gymnospermae and Angiospermae) endemic to China (149 species) accounted for 1.00% of the total species endemic to China. There were 57 species endemic to the Qinghai-Tibetan Plateau, including eight species of Lycopodiophyta, three species of Gymnospermae and 46 species of Angiospermae. The species of seed plants (Gymnospermae and Angiospermae) endemic to the Qinghai-Tibet Plateau (49 species) accounted for 1.30% of the total seed plants endemic to the Plateau.

In terms of protection level, there were 22 species of Class-I protected plants, including two species of Lycopodiophyta, one species of Pteridophyta, nine species of Gymnospermae, nine species of Angiospermae and one species of Cyanophyta. There were 328 species of Class II protected plants, including five species of Bryophytes, 24 species of Lycopodiophyta, 10 species of Pteridophyta, 16 species of Gymnospermae and 273 species of Angiospermae.

In terms of endangered status, one species, *Cystopterischinensis*, was extinct in the wild (EW), 17 species were critically endangered (CR), such as *Calanthedulongensis*, 91 species were endangered (EN), such as *Cephalotaxuslanceolata*, 90 species were vulnerable (VU), such as *Cypripediumhenryi* and 30 species were near threatened (NT), such as Parispolyphyllavar.yunnanensis, 60 species were of least concern (LC), such as *Huperziaselago* and 62 species had data deficiency.

### Geographical distribution pattern

On the Qinghai-Tibetan Plateau, the diversity distribution pattern of national key protected wild plants showed a decreasing trend from the southeast to northwest. There were 169 species and 13 varieties in the Yunnan Province, with 13 Class-I and 156 Class-II species. In Sichuan Province, 169 species, 12 varieties and one subspecies were distributed, including nine Class-I species and 173 Class-II species. In the Tibet Autonomous Region, 155 species and five varieties were distributed, including nine Class-I species and 151 Class-II species. In Gansu Province, 75 species, five varieties and one subspecies were distributed, including six Class-I species and 75 Class-II species. In Qinghai Province, 46 species were distributed, which were all Class-II species. Eighteen species were distributed in the Xinjiang Uygur Autonomous Region, including 18 Class-II species.

**Figure 3.**
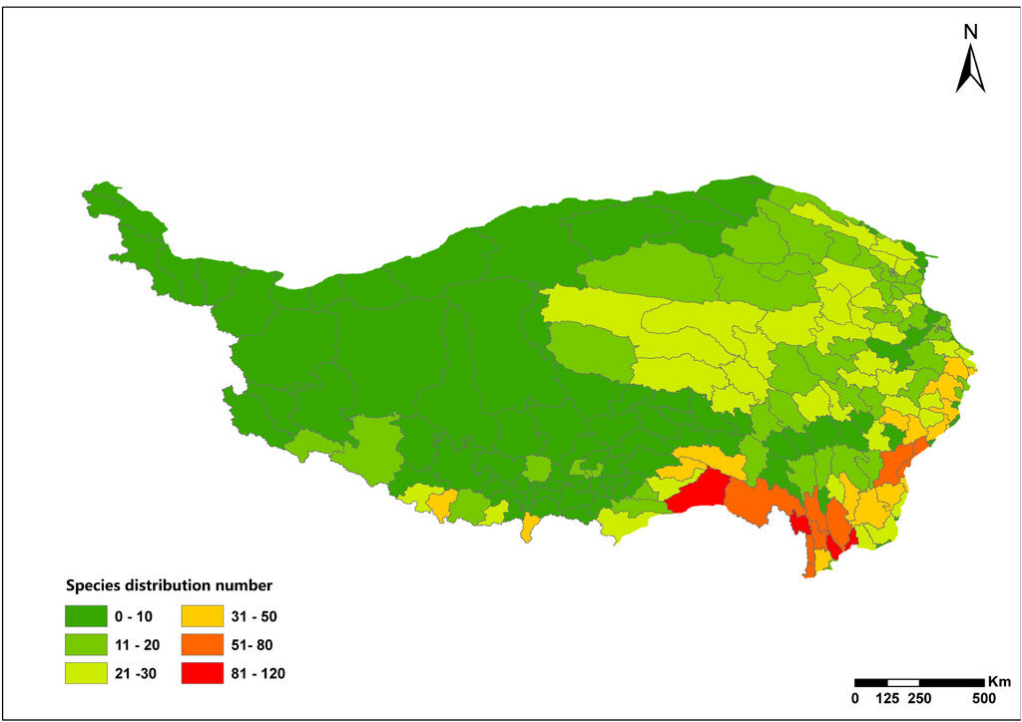
Number of national key protected wild plants on the Qinghai-Tibetan Plateau, based on county-level distribution.

From the distribution at the county-level (Fig. 3), the distribution of nationally protected wild plants on the Qinghai-Tibetan Plateau was the highest in Gongshan and Yulong counties in Yunnan and Motuo County in Tibet, with at least 80 species. Species distribution in Deqin, Weixi and Fugong counties of Yunnan; Chayu County of Tibet; and Kangding, Luding and Tianquan counties of Sichuan was concentrated, with 51–80 species. The species distribution in most areas from southeast to northwest Tibet was very low, with fewer than 10 species.

**Figure 4.**
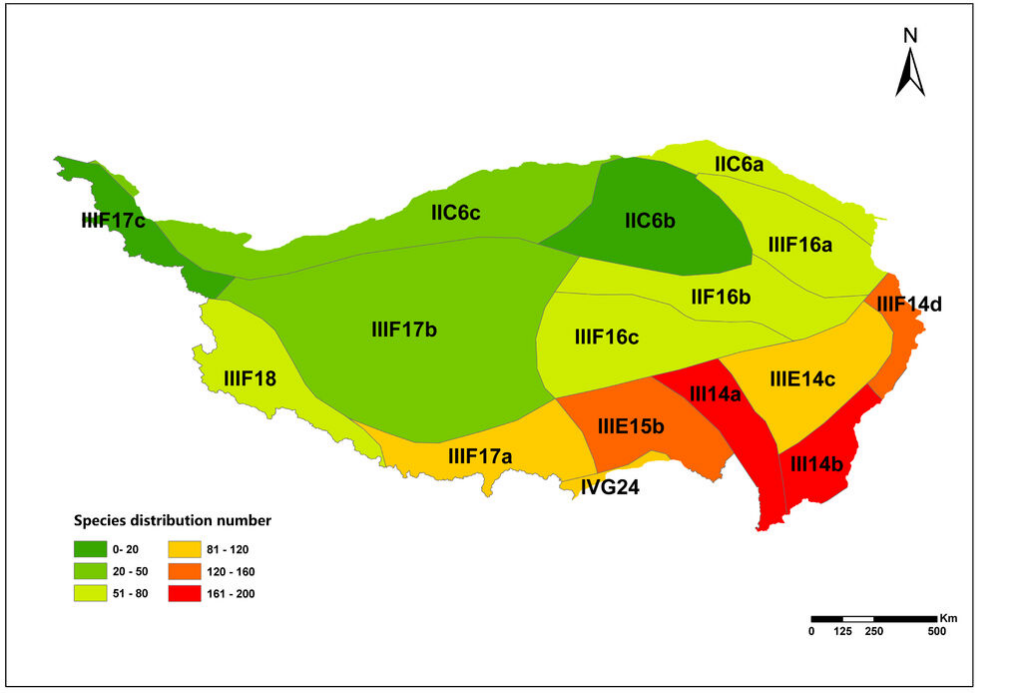
Distribution and quantity of number of national key protected wild plants on the Qinghai-Tibetan Plateau, based on the floristic scale.

From the floristic distribution (Fig. 4), the species distributed in the Sanjiang Valley subregion (IIIE14a) and the South Hengduan Mountain subregion (IIIE14b) were the most dense, with more than 160 species, followed by species distributed in Southeast Asia of Tibet (IIIE15b) and Taohe-Minshan subregion (IIIE14d). Only less than 10 species were found in the Qaidam Basin subregion (IIC6b) and Pamir-Kashi Kunlun subregion (IIF17c).

## Discussion

The Qinghai-Tibetan Plateau is not only a gene pool for plateau species and a hotspot for global biodiversity protection, but also an important ecological security barrier for China and Asia, which is one of the key areas of ecological civilisation construction in China. Due to the fragility and irreversibility of the alpine ecosystem, their environmental sensitivity and the increasing impact of climate warming and human disturbance, their biodiversity is facing serious challenges. Therefore, the protection of biodiversity and ecological security is the core task of ecological protection in the Qinghai-Tibetan Plateau. To date, 196 naturally-protected areas, including national parks, nature reserves, wetland parks, national forest parks and national geoparks, have been designated on the Qinghai-Tibetan Plateau (Hu 2020). These natural protected areas play an irreplaceable role in the protection of biodiversity resources on the Tibetan Plateau, especially in the protection of endangered and critical species on the Plateau (Fu et al. 2021). However, in the literature review, it was found that the population status of national key protected wild plants in this area, including the distribution of wild species, population size and genetic background information, is not yet clear and there are still blind areas of species distribution. Therefore, it was necessary to conduct a comprehensive investigation and assessment of the current status of these protected plants in the field.

Plant diversity patterns play an important role in maintaining the ecological balance of the Qinghai-Tibetan Plateau. Historical orogeny and related climatic changes are the main driving forces behind the formation of plant diversity patterns in the Qinghai-Tibetan Plateau (Muellner-Riehl 2019). On the one hand, the uplift of the Qinghai-Tibetan Plateau has greatly changed the surface pattern of the region and the geographical barriers such as mountains, canyons and "sky island" formed have restricted the gene flow amongst plant populations and promoted species differentiation (Ren et al. 2015, Khan et al. 2018). On the other hand, the uplift of the Qinghai-Tibetan Plateau also leads to the Indian and East Asian monsoons (Tada et al. 2016) and the change in the monsoon climate may be another important driving force for plant evolution in this region (Liu et al. 2013, Wen et al. 2014, Sun et al. 2015). In addition, the alternation of Ice Ages experienced in this region since the Pleistocene has led to habitat fragmentation of many originally continuously distributed species, which retreat to isolated Ice-Age sanctuaries, resulting in the lineage differentiation and the generation of many new species through allogeneic speciation (Wang et al. 2010, Ren et al. 2017). Under the influence of these special natural geographical conditions and speciation mechanisms, most of the national key protected plants on the Qinghai-Tibetan Plateau have a narrow distribution and small population size, which highlights the importance and urgency of sorting, investigating, collecting and protecting these germplasm resources.

In this study, the species distribution information was mainly sorted based on flora, virtual specimens and part of the field investigation data; thus, errors in the data are inevitable. Subsequent research on physical specimens and field investigation data can further clarify the distribution status of national key protected plants on the Qinghai-Tibetan Plateau.

## Supplementary Material

088B7508-AD64-5953-9906-95A227F86971 10.3897/BDJ.11.e103289.suppl1 Supplementary material 1 Checklist of National Key Protected Wild Plants on the Qinghai-Tibetan Plateau
Data type Excel csv spreadsheet File: oo_847978.csv https://binary.pensoft.net/file/847978 Ronglian Chen, Faqi Zhang, Shilong Chen, Xiaofeng Chi
